# Deep Longitudinal Proteomics Profiling Reveals Biological Pathways Responding to GRF6019 in Two AD Clinical Trials

**DOI:** 10.1093/geroni/igab046.016

**Published:** 2021-12-17

**Authors:** Benoit Lehallier, Tibor Nanasi, Jonas Hannestad, Steven Braithwaite

**Affiliations:** R&D, Alkahest, California, United States

## Abstract

Blood has been widely investigated to discover biomarkers and gain insights into the biology of aging and age-related diseases. Its protein composition provides insights into complex biological processes, as proteins are often direct regulators of cellular pathways. In clinical trials, selected proteins have been used as primary and secondary endpoints, but recent methodological developments allow the measurement of thousands of proteins with very high sensitivity and specificity. In two phase 2 clinical trials testing the safety, tolerability, and feasibility of infusions of the plasma fraction GRF6019 in Alzheimer's disease (AD), we measured more than 7000 proteins in plasma over the course of the clinical trials. Differential trajectories analysis revealed groups of proteins and pathways that were responding to GRF6019. Several pathways were relevant to the biology of aging and AD and our study suggests that deep proteomics profiling can inform on specific biological processes responding to treatment in clinical trials.

